# Unusual Presentation of Beckwith-Wiedemann Syndrome in an Extremely Low Birth Weight Infant

**DOI:** 10.7759/cureus.63099

**Published:** 2024-06-25

**Authors:** Abdulla A Alhamdan, Eman S Shajira

**Affiliations:** 1 Department of Pediatrics, Bahrain Defense Force Hospital, Royal Medical Services, Riffa, BHR

**Keywords:** hepatoblastoma, wilms tumor, abdominal wall defect, hemihypertrophy, macroglossia, macrosomia, extremely low birth weight, beckwith-wiedemann syndrome

## Abstract

Beckwith-Wiedemann syndrome (BWS) is a genetic disorder that affects fetal growth in which those afflicted present with features pertaining to that, such as macrosomia, macroglossia, hemihypertrophy, and abdominal wall defects. This case reports the presentation of an infant diagnosed with BWS who was born with an extremely low birth weight of 980 grams, in contrast to the typical presentation of overgrowth and macrosomia. As a result, reaching a diagnosis of BWS was delayed until the patient reached eight months of age, when other clinical features of BWS, such as hemihypertrophy, became apparent on follow-up visits. Although genetic testing can be used to diagnose this condition, a clinical scoring system consisting of a patient’s clinical features is sufficient, allowing for a timely and precise diagnosis, which is of great significance to allow for early screening and detection of the associated embryonal tumors with such a syndrome.

## Introduction

Beckwith-Weidman syndrome (BWS) is a rare genetic syndrome that occurs in one in 10,000 live births [1]. Beckwith-Weidman syndrome’s underlying pathology is secondary to the genomic imprinting of chromosome 11p15, which is a fundamental factor in ensuring adequate intra- and extra-uterine growth of a baby [2, 3].

Furthermore, BWS is characterized by its phenotype heterogenicity; in other words, these patients can present with a wide set of clinical features that vary from patient to patient. The more common features include macrosomia, macroglossia, hemihypertrophy, and abdominal wall defects such as omphalocele or umbilical hernia. Other recognized features include neonatal hypoglycemia, vascular lesions such as hemangioma or naves simplex, and visceromegaly, among other features [2, 3].

Several diagnostic methods for BWS are available. Molecular testing is mandatory in patients with a positive family history of genomic imprinting on chromosome 11p15. If the patient's family history is insignificant and BWS is clinically suspected, a certain scoring system is sufficient to reach the diagnosis if the criteria are fulfilled [4].

It is vital that a prompt diagnosis of BWS be established at the earliest possible time in view of its known associated increased risk of embryonal tumors such as Wilms tumor and hepatoblastoma, which have the highest risk of developing before two years of age. An early diagnosis allows earlier screening and detection of such tumors [1, 2].

We report an unusual presentation of a patient who was born with an extremely low birth weight (defined as those neonates born with a weight of < 1000 g) and diagnosed with BWS at eight months of age.

## Case presentation

A female neonate was born at 28 weeks of gestation through an emergency cesarean section secondary to antepartum hemorrhage. Her APGAR score was three, five, and eight at one, five, and 10 minutes, respectively, and her birth weight was 980 grams. She was admitted to the level III neonatal intensive care unit (NICU) for the management of a very preterm and extremely low birth weight neonate. Her stay was complicated by respiratory distress syndrome, neonatal jaundice, feeding intolerance, and anemia of prematurity. During her stay in the unit, she had a small red spot over the anterior fontanelle that rapidly grew to a 2 x 2 cm non-pulsating hemangioma. An ultrasound of the brain was requested and revealed no intracranial-associated anomalies. An echocardiogram examination was normal, hence the start of oral propranolol. Furthermore, she had a small reducible umbilical hernia and a protruded tongue that raised the suspicion of congenital hypothyroidism; however, her thyroid function profile was normal. Moreover, the presence of these features at the time did not raise suspicion of BWS, as these were common findings associated with premature infants. After the effective management and resolution of her acute problems, she was kept in a level II NICU for feeding and growth and discharged in good condition at a weight of 2.05 kg.

**Figure 1:**
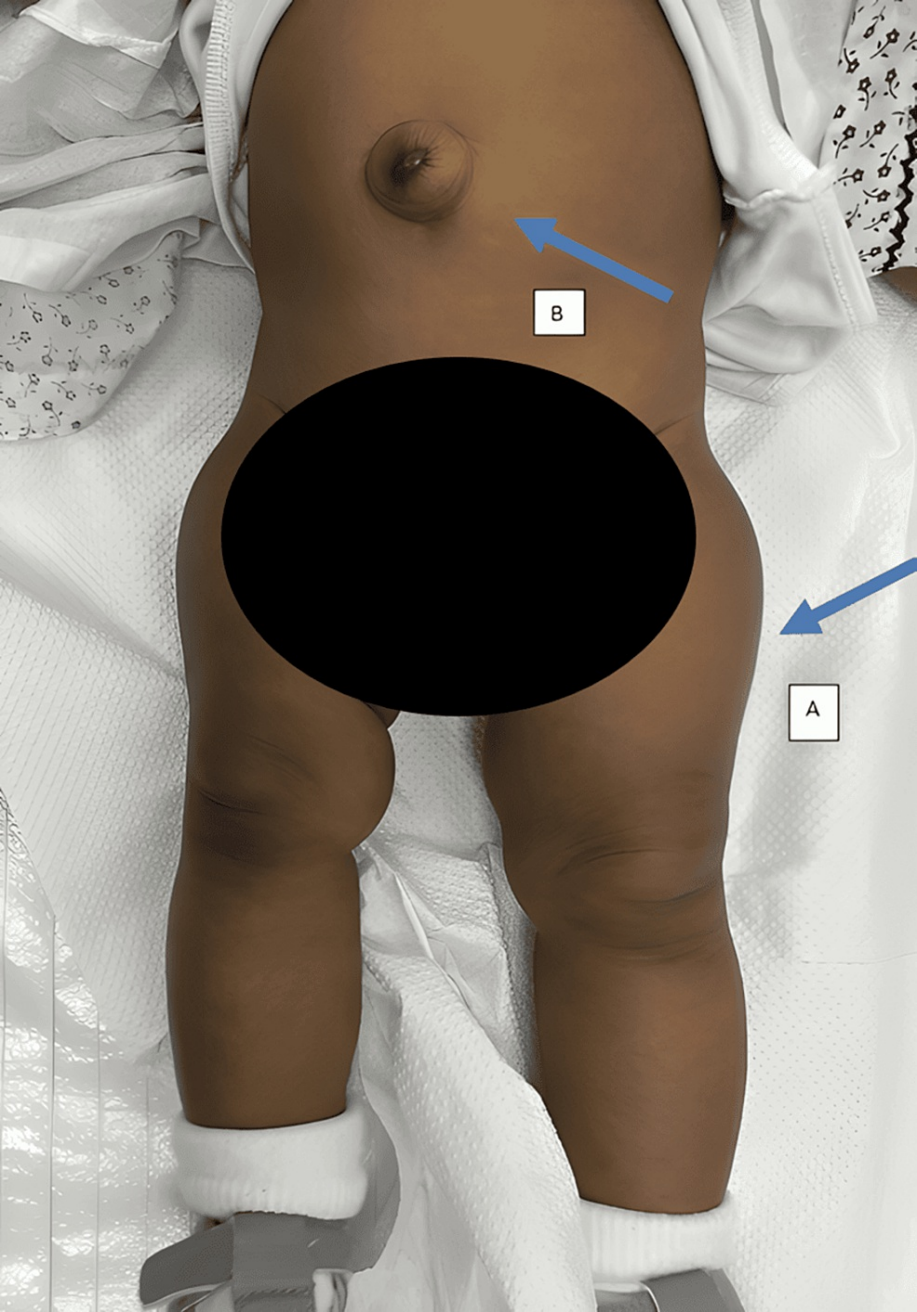
A photograph illustrates some of the findings of Beckwith-Wiedemann syndrome in the patient. A: left-sided hemihypertrophy; B: umbilical hernia

It was not until the age of eight months, during a follow-up visit, that a clear size discrepancy was noticed between the lower limbs. The left side was considerably bigger and longer by approximately 2 cm, as appreciated in Figure 1.

**Figure 2:**
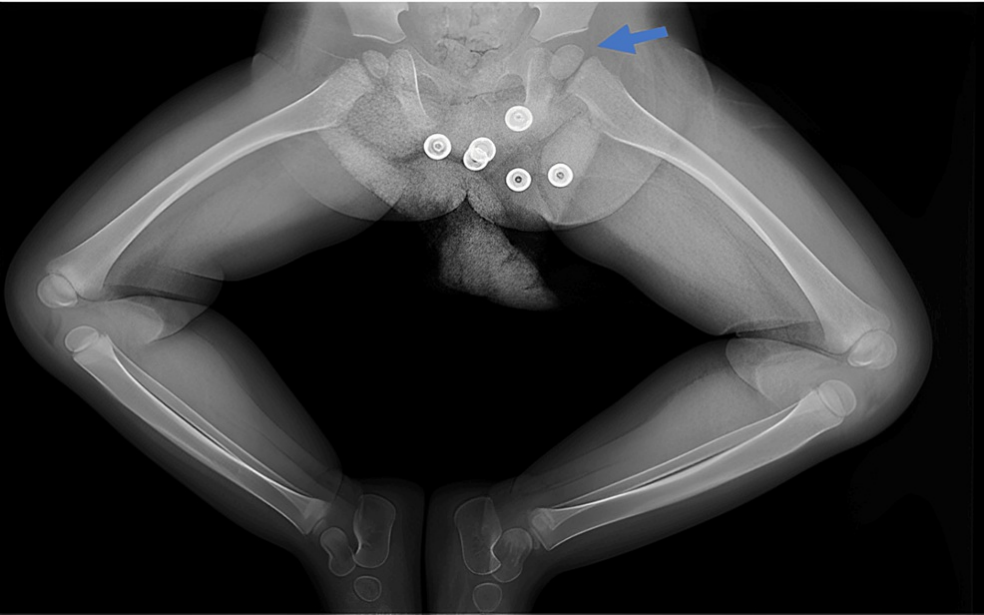
Frog leg view of lower limb demonstrating discrepancy in ossification of proximal femoral epiphysis between right and left bones, with the left being larger.

This was confirmed by an X-ray, which showed a discrepancy in the ossification of the proximal femoral epiphysis between the right and left bones, with the latter being larger (Figure 2).

**Figure 3:**
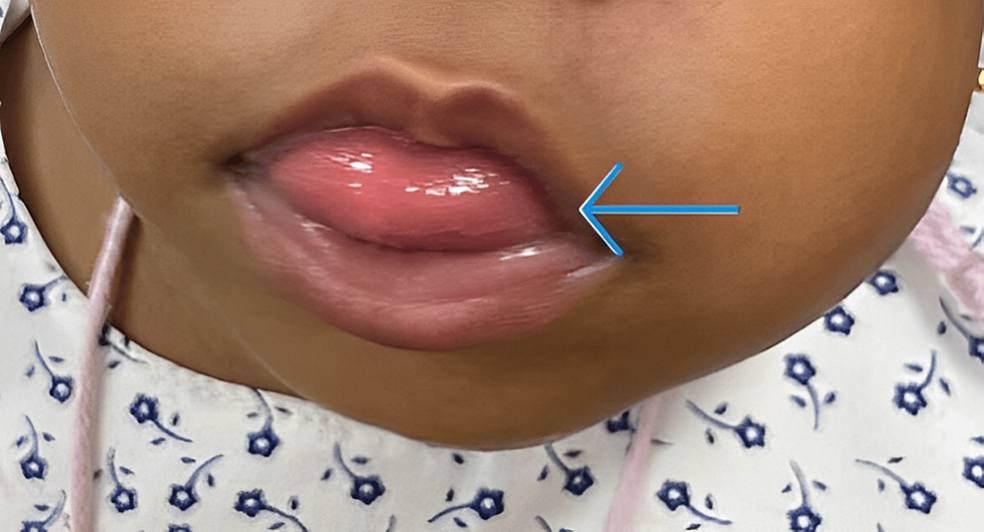
A photograph showing macroglossia in the patient.

**Figure 4:**
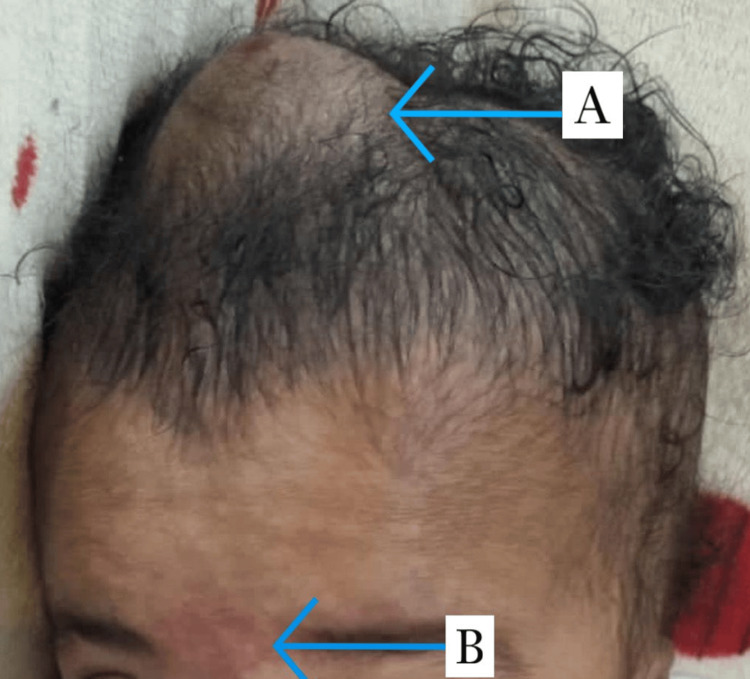
Other findings of Beckwith-Wiedemann syndrome in the patient A: scalp hemangioma; B: nevus flammueus

Therefore, the presence of the left-sided hemihypertrophy accompanying other pre-existing features such as macroglossia, umbilical hernia, nevus flammueus, and hemangioma made the clinical picture of BWS apparent (Figures 3, 4).

The patient was diagnosed with BWS as she fulfilled the criteria for clinical diagnosis using the Beckwith-Weidman spectrum scoring system. The parents were informed and counseled regarding the different aspects of the syndrome. Screening for embryonal tumors through alpha-fetoprotein (AFP) markers and abdominal ultrasounds was started. Both initial results were negative.

The hemangioma size decreased with the effect of propranolol given at 1 mg/kg/day, which was continued for a total of one year. The baby remained clinically stable with normal neurodevelopmental milestones and was growing appropriately according to the growth centiles for her sex and age.

At present, she is being followed up by a multidisciplinary team of pediatricians, endocrinologists, pediatric orthopedicians, and occupational therapists. A pair of special lifting shoes to align the length of her lower limbs was custom-made for her. The effect of the hemihypertrophy on the gross motor aspect of her development will be reassessed in further follow-ups, and the patient will be screened for embryonal tumors every three to four months according to guidelines.

## Discussion

Beckwith-Wiedemann syndrome is a disorder characterized mainly by overgrowth, which usually presents in the neonatal period. A scoring system called Weksberg diagnostic criteria, which encompasses major and minor findings of BWS, has been established in order to aid in the diagnosis. The presence of three or two major and one minor findings supports the clinical diagnosis of BWS [4]. Major criteria include macroglossia, which is prevalent in 97% of these patients, macrosomia in 84%; abdominal wall defects in 80%, hemihypertrophy in 64%, and visceromegaly in 41% of the patients, in addition to other features such as embryonal tumors, a positive family history, outer ear anomalies, kidney and ureter anomalies, and a cleft palate. Minor criteria include neonatal hypoglycemia, prematurity, placentomegaly, nevus flammueus, polyhydramnios, diastasis recti, cardiomegaly or hypertrophic cardiomyopathy, typical facies such as pointed chin and high forehead, polydactyly, supernumerary nipple, and advanced bone age [4,5].

Due to the diverse presentation of BWS-afflicted patients, another scoring system called the Beikwith-Weidman spectrum scoring system has been established to accommodate this fact. Features in this system are classified into cardinal and suggestive symptoms. Each cardinal feature is assigned two points, and each suggestive feature is assigned one point. The presence of four or more points is sufficient to establish a clinical diagnosis. On the other hand, if the patient is assigned two or more points, molecular testing is required to confirm the diagnosis. The presence of less than two points in the patient suggests that BWS is unlikely and genetic testing is not warranted for such cases. Cardinal and suggestive features are illustrated in Table 1 [4].

**Table 1:** Cardinal and suggestive features of the Beikwith-Weidman Spectrum scoring system SDS: standard deviations Source: [4]

Cardinal features (two points for each feature)
Macroglossia
Exomphalos
Lateralized overgrowth
Multifocal and/or bilateral Wilms tumors or nephroblastomatosis
Hyperinsulinism (lasting >1 week and requiring medical treatment)
Pathology findings: adrenal cortex cytomegaly, placental mesenchymal dysplasia, or pancreatic adenomatosis
Suggestive features (one point for each feature)
Birthweight >2SDS above the mean
Facial nevus flammeus
Ear creases and/or pits
Umbilical hernia and/or diastasis recti
Hyperinsulinism (lasting <1 week)
Typical tumors: neuroblastoma, rhabdomyosarcoma, unilateral Wilms tumors, hepatoblastoma, adrenocortical carcinoma, or pheochromocytoma
Nephromegaly and/or hepatomegaly

According to Weksberg's diagnostic criteria, our patient met three of the major criteria: macroglossia, hemihypertrophy, and an abdominal wall defect (umbilical hernia). In addition, the patient fulfilled two of the minors: her being a premature infant and the presence of scalp hemangioma. Furthermore, the patient also fulfilled the criteria for clinical diagnosis according to the Beckwith-Weidman spectrum scoring system, as she fulfilled more than four points (macroglossia, lateralized overgrowth, umbilical hernia, and facial nevus flammeus). It is important to be aware that while BWS resembles an overgrowth syndrome, with macrosomia being a predominant feature in 84% of cases [4], not all patients present with the typical clinical picture. Our patient was an outlier who was born with an extremely low birth weight, which subsequently delayed the diagnosis until clinical features of BWS became evident in subsequent outpatient clinic visits.

As aforementioned, this syndrome is associated with an elevated risk of the development of embryonal tumors in about 8% of cases, with the risk being greatest in the initial two years of life [2]. The most common tumors are Wilms tumor and hepatoblastoma, which occur in 2.5% and 1.7% of these patients, respectively [1, 2]. In view of this, screening is essential once the diagnosis is established to reduce related morbidity and increase survival rates [2].

Once our patient was diagnosed with BWS, a thorough screening protocol was initiated, which included performing abdominal and renal ultrasounds to rule out masses and measuring AFP. Alpha-fetoprotein is a marker with diagnostic relevance for the aforementioned common tumors and should be serially measured every three months until the patient reaches four to eight years of age [1,2]. In the case of our patient, the initial and subsequent screening tests were normal.

Even though the rare presentation of our case made the recognition of BWS challenging, the timely diagnosis was crucial to allow for better counseling of the family about the various aspects of BWS and to initiate the patient into our screening protocol.

## Conclusions

Beckwith-Wiedemann syndrome is a rare genetic syndrome that can present with a multitude of different manifestations, which vary on a case-to-case basis. Examples of these include macrosomia, hemihypertrophy, macroglossia, abdominal wall defects, and so on. Comprehensive scoring systems are available that are efficient in establishing the diagnosis. In our case, it was challenging given the fact that the patient presented as an extremely low birth weight baby instead of the usual presentation with macrosomia. Therefore, it is important to consider that not all cases of BWS present the same, and although many patients have similar features, there are some with their unique presentation. Finally, establishing diagnosis early is of great significance to carry out screening for the associated tumors and prevent any complications that may arise with this syndrome.
